# Save the Babies

**Published:** 1876-07

**Authors:** 


### *SAVE THE BABIES.*

During these early days of July, with the thermometer in the nineties, how often have we lamented our inability to rescue from death every one of the tens of thousands of helpless babes whose lives are soon to be blotted out amid agony and suffering, for which parents and physicians are responsible. We have plead for these helpless little ones during the past ten years, and shall continue to plead for them while we live. Of all the diseases that afflict children, the most trivial and harmless when properly treated, or when not treated at all, are Cholera Infantum, and what are erroneously called "bowel troubles" of children. It is the "treatment,"

not the disease, that destroys the little patients in ninety-nine out of every hundred fatal cases; and the one thing that accomplishes the mi schief is opium.

Hardly a week passes that the newspapers do not notice some case of poisoning by "soothing syrup;" and who believes that one such case in a thousand is ever reported? Any physician who would prescribe opium in any form to a child, for any disease, should be driven from the profession with the "cat-o-nine-tails." We will repeat what we have so often said before in relation to "Summer Complaint" of children. "Colera Infantum" differs from other so-called bowel troubles of children only in degree. It is "Summer Complaint" in a severer form. We have never seen a case of this kind where there was any actual disease of the bowels. Still there is not one physician in a thousand who, when called, will not pronounce such a case a disease of the bowels, and prescribe opium in some form. It would generally be a mercy to the child, and save it much unnecessary suffering, if he were to bring a revolver and kill the patient by a more rapid, but not less certain process. These bowel troubles of children do not indicate disease, but simply a torpid condition of the liver, induced by the irritation of teething, by cold, or by improper, or insufficient nourishment. Where a child can have proper care, promptly, there is no danger of a fatal result. The child should be kept in a cool, quiet, comfortable place, and dressed in clothing appropriate to the weather. A flannel bandage should be worn about the body. Concentrated foods, a little at a time, should be given, and ice, instead of water. Two or three teaspoonsful of brandy toddy may be given every hour or two, with good effect. If the child does not rapidly recover, medicine should be given to act gently on the liver. This will gradually check the discharges from the bowels, by inducing healthy action of the liver. To check them in any other way is to endanger the life of the child, as the pent up fluids are certain to be forced into the lungs and brain, when the discharges from the bowels are checked by opium. The anxious mother is always alarmed on account of these frequent passages, and the physician foolishly prescribes the fatal drug to quiet her fears, and the child suddenly dies of congestion, when she believed it out of danger.

Since this article will be read for the purpose of gaining information on the subject which we are discussing, it would be unfair to withhold any advice which might be the means of doing good, from fear that our motives might be misjudged or minunderstood. We will therefore say what we deem it our duty to say without further explanation or apology.

Children suffering from "summer complaints," will almost always recover with good nursing alone. But the progress of recovery is usually slow, and the discharges from the bowels debilitate so that the little patient may die from exhaustion, if there is an unfavorable change in the symptoms. It is best always to be on the safe side. Children will not die of "Summer Complaint," "Cholera Infantum," or any diseases incident to teething, when the treatment is properly directed to the true seat of the disease, which is the liver. It is not necessary to confine ourselves to any particular prescription or medicinal preparation so long as the desired result is accomplished; but we never could see any reason for condemning a medicine simply because it is extensively advertised. We have therefore, for several years past, prescribed "Van Deren's Remedy," for all bowel complaints of children. It is prepared by Hall & Ruckel, wholesale druggists, No. 218 and 220 Greenwich Street, New York. They send a package by mail to any address, on receipt of 50 cents.

No family where there are children should be without this invaluable medicine for a day. A lady recently wrote to us that she had purchased a package of "Van Deren's Remedy" on our recommendation, and asked if it would be safe to give it? In order to save others the trouble of further inquiries, we will say that "Van Deren's Remedy" is put up in the form of little sugar granules, and that, although its action is very prompt and decisive, and has always cured every case of Cholera Infantum within twenty-four hours when we have given it, still, it would do no harm if a child were to take the entire contents of the vial at once. It contains no opium, and nothing that could possibly do harm. It is intended to do good only, and that it always does. Do not hesitate to give it according to directions.

